# Molecular profile reveals immune-associated markers of medulloblastoma for different subtypes

**DOI:** 10.3389/fimmu.2022.911260

**Published:** 2022-07-28

**Authors:** Jinyi Chen, Zhuang Kang, Shenglan Li, Can Wang, Xiaohong Zheng, Zehao Cai, Lexin Pan, Feng Chen, Wenbin Li

**Affiliations:** ^1^ Department of Neuro-oncology, Cancer Center, Beijing Tiantan Hospital, Capital Medical University, Beijing, China; ^2^ School of Mechatronical Engineering, Beijing Institute of Technology, Beijing, China

**Keywords:** MethylCIBERSORT, immune infiltration, WGCNA, PPI, medulloblastoma

## Abstract

Medulloblastoma, a common pediatric malignant tumor, has been recognized to have four molecular subgroups [wingless (WNT), sonic hedgehog (SHH), group 3, group 4], which are defined by the characteristic gene transcriptomic and DNA methylomic profiles, and has distinct clinical features within each subgroup. The tumor immune microenvironment is integral in tumor initiation and progression and might be associated with therapeutic responses. However, to date, the immune infiltrative landscape of medulloblastoma has not yet been elucidated. Thus, we proposed MethylCIBERSORT to estimate the degree of immune cell infiltration and weighted correlation network analysis (WGCNA) to find modules of highly correlated genes. Synthesizing the hub genes in the protein–protein interaction (PPI) network and modules of the co-expression network, we identify three candidate biomarkers [GRB2-associated-binding protein 1 (GAB1), Abelson 1 (ABL1), and CXC motif chemokine receptor type 4 (CXCR4)] *via* the molecular profiles of medulloblastoma. Given this, we investigated the correlation between these three immune hub genes and immune checkpoint blockade response and the potential of drug prediction further. In addition, this study demonstrated a higher presence of endothelial cells and infiltrating immune cells in Group 3 tumor bulk. The above results will be conducive to better comprehending the immune-related pathogenesis and treatment of medulloblastoma.

## Introduction

Medulloblastoma (MB) is considered to be a highly malignant and fast-growing brain tumor among pediatric central nervous system (CNS) malignancies, accounting for 8%–10% of all pediatric brain tumors and is becoming the most common type (1). Classification of MB was primarily based on histological features, age at diagnosis, tumor resection range, and metastasis, while with recent developments in high-throughput transcriptome data analyses, the subclassification of MBs applied by a transcriptional approach can be achieved (2, 3). The international consensus destination of MB distinguished by advanced genomic research was named as follows: wingless-activated (WNT-MB), sonic hedgehog-activated (SHH-MB), Group 3, and Group 4, each characterized by unique genetic alterations, transcription al/methylation profiles, and clinical outcomes (4–7). These four working groups are now considered to be individual biological entities.

WNT-MBs are the most well-known subgroup of MB with an activation of the WNT pathway and harbor mutations in exon 3 of CTNNB1 and monosomy chromosome 6. Indeed, the long-term survival rates for patients with WNT-MBs are likely to exceed 90%; most patients often die as a consequence of complications of therapy or secondary neoplasms rather than from recurrent WNT-MBs (8). SHH-WBs, with an activation of the SHH pathway, have largely been distinguished based on transcriptional profiling. Immunohistochemical staining for SFRPI, or GRB2-associated-binding protein 1 (GAB1), and deletion of chromosome 9q are other approaches to distinguish SHH-MBs. The overall prognosis of SHH-MBs is similar to that of Group 4-MBs, between the WNT-MBs (good) and Group 3-MBs (poor) (4). Survival among SHH-MB patients was significantly worse for combined with chromosome loss (such as chromosome 3p, 10q, and 17p) and PTCH1 mutations (9). TP53 mutational status indicates distinct outcomes in SHH-MBs: TP53 wild type tumors are more frequent among adults and young children and are related to a favorable prognosis, while TP53 mutation tumors are common among older children and are linked to adverse outcomes (10). Unlike WNT-MBs and SHH-MBs, group 3 and group 4 are not related to well-defined activated signaling pathways. Group 3 has a high metastatic rate that implies poorer prognoses particularly in those with amplified MYC (11, 12). Structural aberration such as loss of 16q, 10q, and 9q and gain of 7 and 1q is most recurrent in group 3-MBs (13). Group 4-MBs are the most prevalent type of MB, but their molecular pathogenesis is not well understood (14). Patients with absent chromosome 11 have an excellent clinical outcome (exceeding 90%). While compared with WNT- or SHH-MBs, adults with Group 4-MBs have a significantly worse prognosis (15). These subgroups with specific genetic alterations and clinical outcomes indicated that tumors with similar transcriptomes may behave in a similar biological manner, providing direction for molecular targeted therapy and clinical risk stratification.

The tumor immune microenvironment (TIME) is of major importance to the evolution of tumors and can modulate the response to chemotherapy and radiotherapy (16). However, current immune therapies, including cancer vaccinations, chimeric antigen receptor T cell (CAR-T) therapy, and immune checkpoint inhibitors (ICIs), do not benefit all patients (17). Targeted therapy has not yet been implemented based on the classification of MBs. Moreover, current chemotherapy is used for post-radiotherapy maintenance and is poorly tolerated by adults and children (especially adults) (18). Therefore, we explored the interaction between TIME and tumors, aiming to provide the basis for taking full advantage of the potential of immune-based therapeutic strategies (19). Previous results that were based on preclinical animal models or an immunohistology profiling of tumors are limited in sample size and struggle to meet statistical requirements. Furthermore, the existing markers for MB subgroup classification are not highly indicative of immune infiltration. With the contribution of high-throughput data analysis, we analyzed large gene expression datasets of MB, including one dataset of 763 tumor samples (20, 21), and dissected immune cell infiltration through MB. In this study, we applied the deconvolution algorithm to analyze the tumor infiltrating immune cells among MB subgroups. We analyzed the differentially expressed immune genes and explored the functional analysis among the MB subgroups, aiming to reveal the immune infiltrative cell distribution and functional pathway enrichment. We then adopted the WGCNA to construct a co-expression network and identify three hub genes according to the immune infiltration of MB. Subsequently, we explored the potential of hub genes for immune checkpoint blockade response and drug prediction. In our study, different subtypes of immune infiltrating cells and immune-related genes were screened for the first time to fully reflect the TIME of MB. Our results aimed to promote immunotherapy and individualize the treatment for MB.

## Materials and methods

### Patients and data collection

All MB gene expression profiles were acquired from the Gene Expression Omnibus (GEO) database and NCBI’s publicly available genomics database (20). Dataset GSE85218 contains DNA methylation and gene expression profiling of primary MB samples from the same public cohort. Gene expression profiling of MBs was from dataset GSE85217 across 763 primary samples based on the platform GPL22286. DNA methylation profiling of 763 primary samples was from dataset GSE85212 based on platform GPL13534. Both GSE85217 and GSE85212 are subseries belonging to the superseries GSE85218. Those 763 intertumoral heterogenic primary samples comprise four distinct entities: WNT (n = 70), SHH (n = 223), Group 3 (n = 144), and Group 4 (n = 326). The immune gene profiles were downloaded from the ImmPort database (http://www.immport.org/, 36 Notes released, September 2020) and the InnateDB (http://www.innatedb.ca/, Version 5.4), which contained 2,533 immune genes.

### Analysis of tumor-infiltrating immune cells among medulloblastoma subgroups

CIBERSORT is a support vector regression modeling to gene expression microarray data, developed to permit the *in silico* deconvolution of complex cellular mixtures and to estimate tumor purity. MethylCIBERSORT is a CIBERSORT-based deconvolution to the DNA methylation profile of tumor tissue and is also able to estimate the degree of immune cell infiltration. Analysis of variance was used to compare the degree of immune cell infiltration among different subgroups and to identify the differential immune infiltrating cells. p < 0.05 was considered to be statistically significant.

### The differentially expressed immune genes among medulloblastoma subgroups

Combined with the immune gene profile, the limma package in the R software was applied to analyze differential gene expressions (|FC| >1.5, adjusted p-value <0.05) in order to screen the target immune gene [differentially expressed immune genes (imm-DEGs)] among the four subgroups of MB. MB is heterogeneous among subgroups. Aiming to screen the unique differential genes of each subgroup, we compared every single subgroup with the remaining three groups and then took the intersection. The differences in analysis results were visualized by using the ggplot2 and pheatmap package in the R software. Gene Ontology (GO) and Kyoto Encyclopedia of Genes and Genomes (KEGG) enrichment analysis were carried out using the clusterProfiler package in the R software in order to annotate the biological function of imm-DEGs (adjusted p-value <0.05 as statistically significant).

### Co-expression network construction

Weighted correlation network analysis (WGCNA) is a powerful algorithm that can be used for uncovering highly correlated genes with similar expression patterns, aiming to identify modules and genes related to disease phenotypes and therapeutic targets. The co-expression network was constructed using the WGCNA package (v.1.69) in the R software (22) and identified the genes with similar expression patterns. The co-expression network construction procedure included the following main steps: 1) define the co-expression similarity matrix; 2) transform the co-expression similarity matrix into the adjacency matrix by a thresholding procedure; 3) use topological overlap measure for network interconnectedness to transform the adjacency matrix into a topological overlap matrix (TOM); 4) perform hierarchical clustering to identify gene modules with a method of constant-height cut based on topological overlap matrix (TOM) dissimilarity. The key modules were gathered using the genes with similar expression patterns under the setting of 3 for soft threshold power, 0.25 for the cut height, and 30 for the minimum module size.

### Identification and correlation analysis of hub genes

The threshold for hub genes was a gene significance (GS) >0.4 and module membership (MM) >0.6 based on the module constructed by a co-expression network with WGCNA. Meanwhile, the protein–protein interaction (PPI) network was constructed by applying the Search Tool for the Retrieval of Interacting Genes (STRING, https://string-db.org/) database. Genes that have a degree of nodes in the PPI network ≥5 were defined as hub genes of the PPI network. Cytoscape software was applied to visualize the PPI network. VennDiagram package in the R software was employed to compare the hub genes of modules with that of the PPI network. Then, corrplot and ggplot2 packages in the R software were also utilized to conduct a relevant analysis of the immune hub gene and the differential immune infiltrating cells.

### Subgroup marker identification based on hub genes

The subgroup marker was identified based on the immune hub gene expression pattern of the four MB subgroups and then combined with the DNA methylation profiles to investigate the difference in the methylation level of the immune hub genes among the four subgroups.

### Validation of subgroup markers

Dataset GSE37418 contained 73 MB samples that conclude 10 samples of SHH, 8 samples of WNT, 16 samples of Group 3, and 39 samples of Group 4, downloaded from the GEO database. The expression patterns of the subgroup markers among the 4 subgroups were then compared and analyzed.

### Construction of the multifactorial regulatory network based on the hub genes and the database

The multifactorial regulatory network that consists of microRNA (miRNA), long-noncoding RNA (lncRNA), and transcriptional factor (TF) was established by combining the hub genes with public databases such as the starBase and Harmonizome. miRNA–messenger RNA (mRNA) and lncRNA–mRNA interaction network was applied by the starBase (http://starbase.sysu.edu.cn/, version 3.0), while mRNA–TF interaction network was applied by Harmonizome (http://maayanlab.cloud/Harmonizome/) database.

### Immunochemistry staining

CD4, CD31, S100A4, PRG2, GAB1, Abelson 1 (ABL1), and CXC motif chemokine receptor type 4 (CXCR4) staining was performed with formalin-fixed, paraffin-embedded MB tissue microarrays. There are five samples in each group (SHH, WNT, Group 3, Group 4). The details of the subgroups and the patients for the tissue microarray were shown in **Supplementary Data**. Tissue microarrays were deparaffinized, rehydrated, and washed three times with phosphate buffered saline (PBS). After performing antigen retrieval by soaking in sodium citrate (pH6.0) and heating to 100°C for 15 min in the microwave oven, the tissue microarrays cooled to ambient temperature. Tissue microarrays were incubated with 3% hydrogen peroxide-methanol for 15 min and 10% goat serum with appropriate Triton X-100 in PBS for 30 min for the sake of blocking the endogenous peroxidase and nonspecific binding, respectively. They were incubated with CD4 (1:150), CD31 (1:1,500), S100A4 (1:250), PRG2 (1:100), GAB1 (1:500), ABL1 (1:100), and CXCR4 (1:400) antibodies overnight at 4°C and then washed with PBS and incubated with the appropriated secondary antibodies for 1 h at ambient temperature. After being stained with 3,3'-Diaminobenzidine (DAB) and counterstained with hematoxylin, the tissue microarrays were mounted and analyzed by inverted microscopy (Zeiss Vert.A1, Germany).

### Correlation between the hub genes and immune checkpoint molecules

We enrolled the genes of immune checkpoints to explore the relationship between the hub genes and the immune checkpoints. Correlation coefficients were evaluated by Spearmen correlation analysis. Corrgram package in R software was applied to demonstrate the correlation between the hub gene expression and the immune checkpoint molecules in each MB subgroup.

### Correlation between the hub genes and IC50 of the targeted drug

We downloaded the response data of 192 antitumor drugs on 1,000 human cancer cell lines from the Genomics of Drug Sensitivity in Cancer (GDSC) database (www.cancerRxgene.org). Correlation coefficients were supported by Spearman’s analysis. The prediction was done using the pRRophetic package of the R software. Ggplot2 package of the R software was used to analyze the correlation between the hub gene expression and IC50 of the antitumor drugs by box diagrams.

### Statistical analysis

All statistical analyses and graph generation were conducted with SPSS 23.0 (IBM, Armonk, NY, USA) and R software (R version 3.5.3; https://www.r-project.org/). Differences among groups were compared with one-way ANOVA. A p-value <0.05 was considered to be statistically significant.

## Results

### Differences of immune cell infiltration among medulloblastoma subgroups

**Figure 1:**
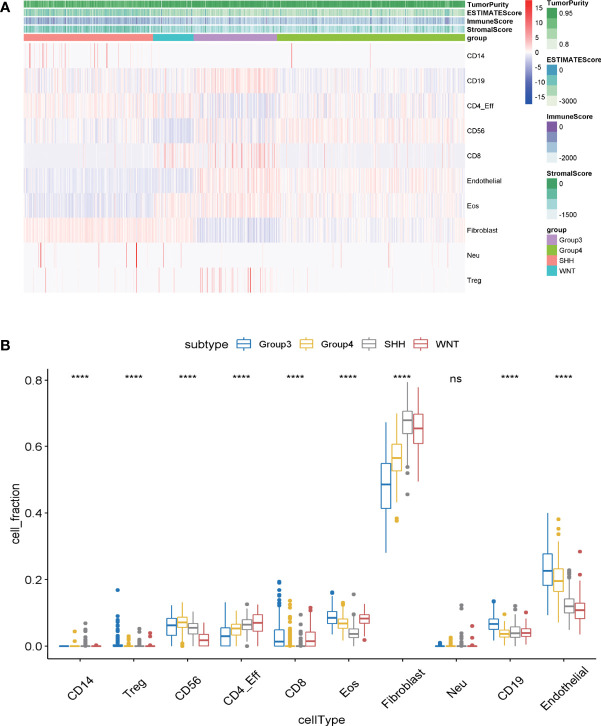
**(A)** The infiltration degree of infiltrating cells in medulloblastoma samples was evaluated by MethylCIBERSORT deconvolution. The abscissa represents medulloblastoma samples, and the ordinate represents the cells existing in tumor samples for identification. **(B)** The differences of immune cell infection among the four subgroups of medulloblastoma were analyzed by one-way ANOVA. Abscissa: the infiltrating cells identified in tumor samples; ordinate: the proportion of each type of cell in tumor tissue. ns, p > 0.05; ****p ≤0.0001.

Tumor-infiltrating immune cells in MB samples were examined with MethylCIBERSORT package in the R software based on the methylation data (23). Based on the transcriptome expression data of tumor samples, the estimate score, immune score, and stromal score are estimated by using estimates according to the proportion of stromal and immune cells, which are used to predict tumor purity. The higher the estimated score, the lower the purity of the tumor. The degree of immune cell infiltration, the immune scores, and the purity of tumors of 763 samples in GSE85212 are displayed in **Figure 1A**. It was evaluated by transcriptomic data within the same group of the samples from GSE85212. Variance analysis was employed to compare the degree of immune cell infiltration among different subtypes of MB and to establish the differential infiltrating immune cells. Results indicated that among the four subgroups of MB, nine kinds of infiltrating immune cells (such as CD14, Regulatory T (Treg), CD58, CD8, and CD19) were statistically significant in the degree of tumor infiltration (**Figure 1B**) (p < 0.001).

### Differentially expressed immune gene and functional analysis among medulloblastoma subgroups

**Figure 2:**
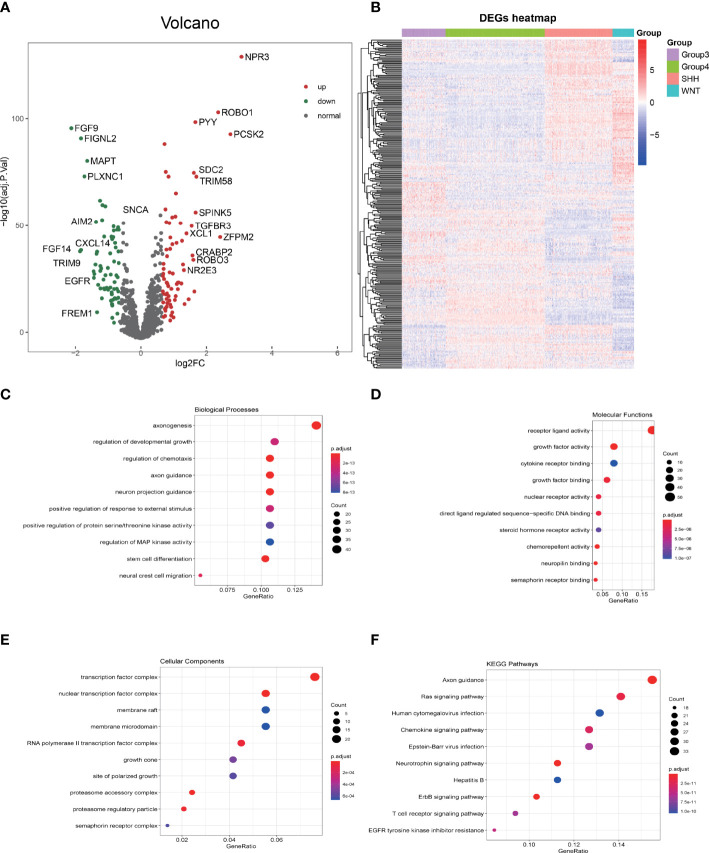
GO and KEGG enrichment analysis of differentially expressed immune genes (imm-DEGs). **(A)** The distribution of differentially expressed genes in group 3 *vs*. group 4 is depicted by the volcano plot. **(B)** The imm-DEGs clustered in the four subgroups of medulloblastoma. **(C)** Imm-DEGs were enriched in the biological processes. **(D)** Molecular functions of Gene Ontology. **(E)** Cellular components of Gene Ontology. **(F)** Kyoto Encyclopedia of Genes and Genomes (KEGG) enrichment analysis of imm-DEGs.

Merging the immune gene sets from ImmPort and InnateDB databases, in total, 2,533 immune-related genes, consisting of 1,794 immune genes from ImmPort immune gene set and 1,052 immune genes from InnateDB immune gene set, were included. Limma, a package for analyzing the differential expression, was utilized to screen the imm-DEGs among the four subgroups of MB. A total of 293 immune genes that expressed differentially among the four subgroups of MB were established (adjusted p-value <0.05). The differential gene distribution and heatmap of differentially expressed genes (DEGs) are illustrated in **Figures 2A, B**.

The biological function enrichment analysis, including GO analysis and KEGG pathway enrichment analysis, was utilized. The GO analysis results emphasized that 293 imm-DEGs were associated with the regulation of chemotaxis, regulation of protein serine/threonine kinase activity, and Mitogen-activated protein (MAP) kinase activity in the BP category (**Figure 2C**); receptor–ligand activity, growth factor binding, and cytokine receptor binding in the MF category (**Figure 2D**); and transcription factor complex, nuclear transcription factor complex, and membrane microdomain in the CC category (**Figure 2E**). In the KEGG pathways, RAS signaling pathway, Human cytomegalovirus infection, Chemokine signaling pathway, and T-cell receptor signaling pathway were all implicated (**Figure 2F**).

### Construction of the co-expression network based on differentially expressed immune genes and identifying key modules

**Figure 3:**
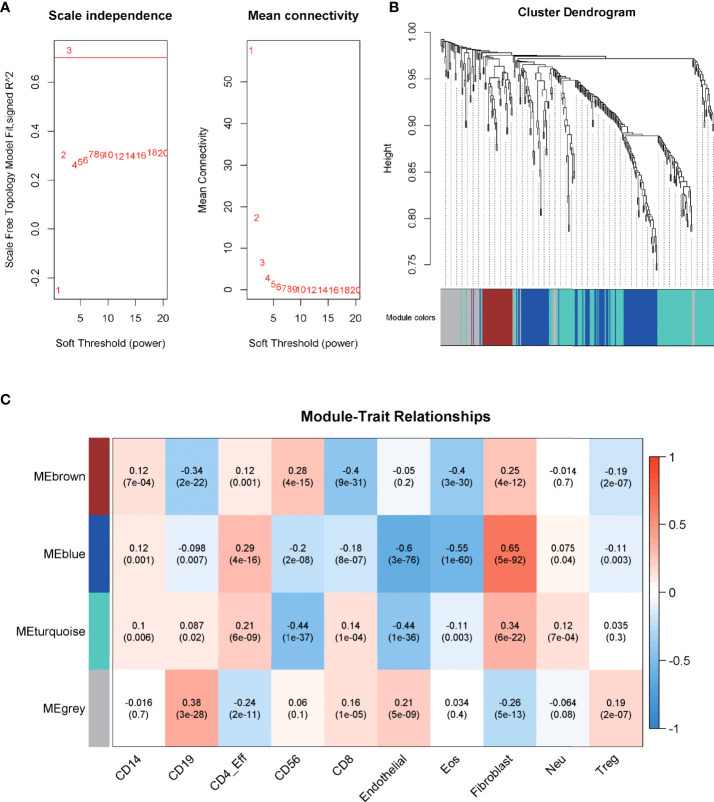
Construction of the co-expression network. **(A)** The parameters for the scale-free topology criterion. **(B)** Cluster the genes into different modules with different reprehensive colors among different modules. **(C)** The degree of immune cell infiltration of samples was collected, and module–trait relationships were calculated according to the correlation.

WGCNA, based on the imm-DEG expression profiles, was employed to construct the co-expression network and distinguish the gene with a similar expression pattern. The parameters of the scale-free topology criterion (**Figure 3A**) were used by setting the soft threshold power to 3, cut height to 0.25, and the minimum module size to 30 to cluster the gene into different modules with different reprehensive colors. In the end, four gene modules were established (**Figure 3B**).

The degree of immune cell infiltration of samples was collected as a trait, and module–trait relationships were calculated according to the correlation in order to match the modules to their strongly related traits. As shown in **Figure 3C**, the blue module was significantly related to Fibroblast cells, Eosinophils (Eos) cells, and Endothelial cells, which are important components of the immune microenvironment, and their interaction with tumor cells plays an important role in the growth of cancer (corr = 0.65, 0.55, -0.6; p = 5e-92, 1e-60, 3e-76, respectively), and the correlation was stronger than that of other modules. Thus, blue was chosen to be the key module.

### Hub gene recognition and the relationship with immune cell infiltration

**Figure 4:**
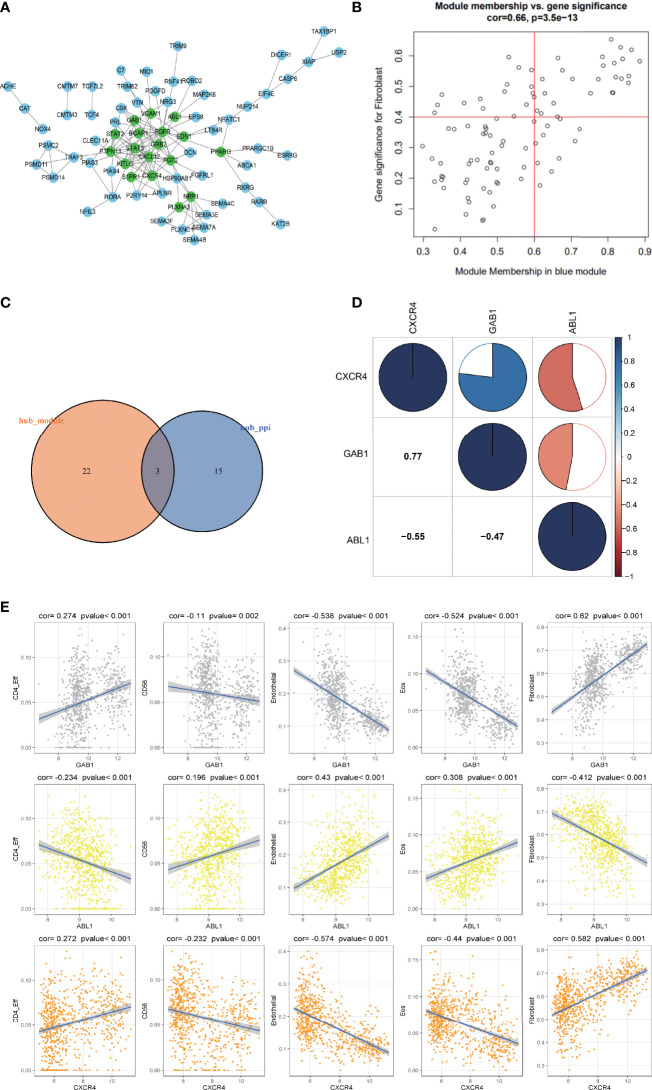
The correlation analysis of the hub gene and its relationship with different immune infiltrating cells. **(A)** The protein–protein interaction (PPI) network was constructed by genes in the blue module. The nodes with a green color on the PPI network represent the hub genes. **(B)** The distribution of gene significance (GS) and module membership (MM) in the blue module. **(C)** The intersection of the immune hub gene set of the PPI network (node degree value ≥5) and the hub gene set of blue modules (GS >0.4, MM >0.6). **(D)** Correlation analysis of the four hub genes. Blue represents a positive correlation, and red represents a negative correlation. The larger the sectorial area of the pie graph, the higher the correlation between the two genes. **(E)** The correlation between hub genes (GAB1, ABL1, CXCR4) and immune infiltrating cells (CD4_Eff, Fibroblast cells, Endothelial cells, and the Eos cells).

Genes from the selected blue key module were used to construct the PPI network, in which 18 hub genes were established by setting the node degree value at ≥5 (**Figure 4A**). Twenty-five candidate hub genes from the blue module were identified by setting the GS >0.4 and MM >0.6 (**Figure 4B**). Three immune hub genes (GAB1, ABL1, CXCR4) were obtained by synthesizing the hub genes in the PPI network and the candidate hub genes in the blue module (**Figure 4C**).

The corrplot package in the R software was employed to investigate the correlation within the 3 hub genes. Results indicated that hub gene GAB1 was positively correlated with CXCR4 (Pearson corr = 0.77); ABL1 was negatively correlated with both CXCR4 and GAB1 (Pearson corr = -0.55, -0.47, respectively); these three hub genes were related to each other (**Figure 4D**).

The correlation between the three hub genes and the differential immune infiltrating cells was also scrutinized. Since the three immune hub genes were from the fibroblast-related module, we explored the correlation between the three hub genes and the marker genes of the cancer-associated fibroblasts (CAFs) (24). Both the hub genes GAB1 and CXCR4 were positively correlated with the CAF marker genes (including ACTA2, VIM, PDPN, and FAP), while ABL1 was negatively correlated with the CAF marker genes (**Supplementary Figure S2**). As for other immune cells, both hub genes GAB1 and CXCR4 have a significant positive correlation with CD4_Eff cells (p < 0.01) and a negative correlation with Endothelial cells and Eos cells (p < 0.001), while hub gene ABL1 has an opposite trend (**Figure 4E**). In addition, we selected the characteristic markers of these correlation immune cells, and the immune hub genes and immune cells were confirmed by immunohistochemistry (**Supplementary Figure S1**). Due to the limited number of tissue samples, we will accumulate more samples for verification in the future.

### Medulloblastoma subgroup marker recognition based on hub genes

**Figure 5:**
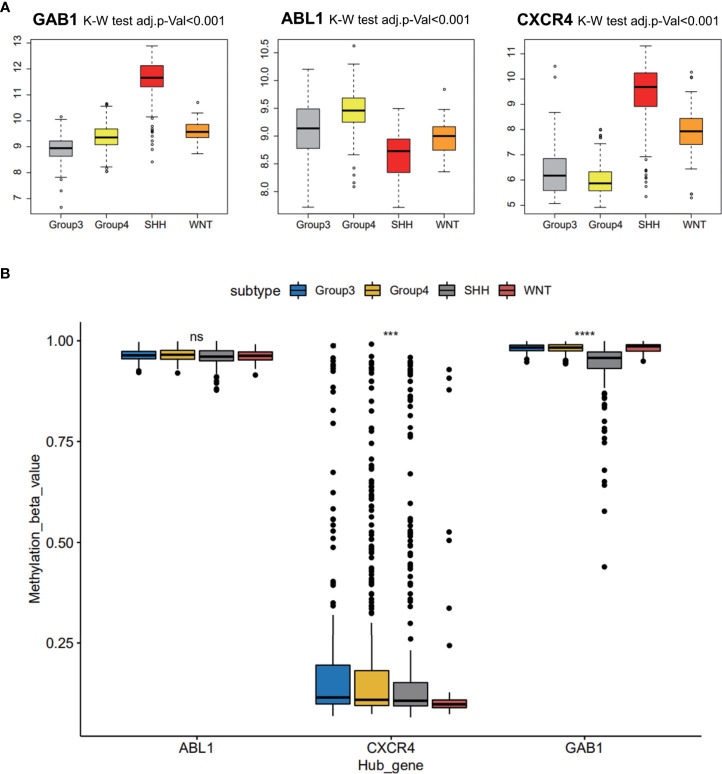
Medulloblastoma subgroup marker recognition based on hub genes. **(A)** Three hub gene expression patterns in the subgroups of medulloblastoma. **(B)** The methylation level of immune hub genes among the four subgroups was established by combining the immune hub gene with the DNA methylated data. ns, p > 0.05; *** p ≤ 0.001; **** p ≤ 0.0001.

Three hub gene expression patterns in the subgroups of MB were also studied, and markers of the subgroups were recognized. It was indicated that immune hub genes GAB1 and CXCR4 were expressed higher in the SHH subgroup than in the three other subgroups; immune hub gene ABL1 was expressed weakly in the SHH subgroup, while it was expressed strongly in the three other subgroups (**Figure 5A**). The methylation level of immune hub genes among the four subgroups was established by combining the immune hub gene with the DNA methylated data. The results of one-way ANOVA showed that the methylation levels of GAB1 and CXCR4 were significantly different among the four subtypes (p < 0.001) (**Figure 5B**).

### Further verification of hub genes in the independent data set and at the histological level

**Figure 6:**
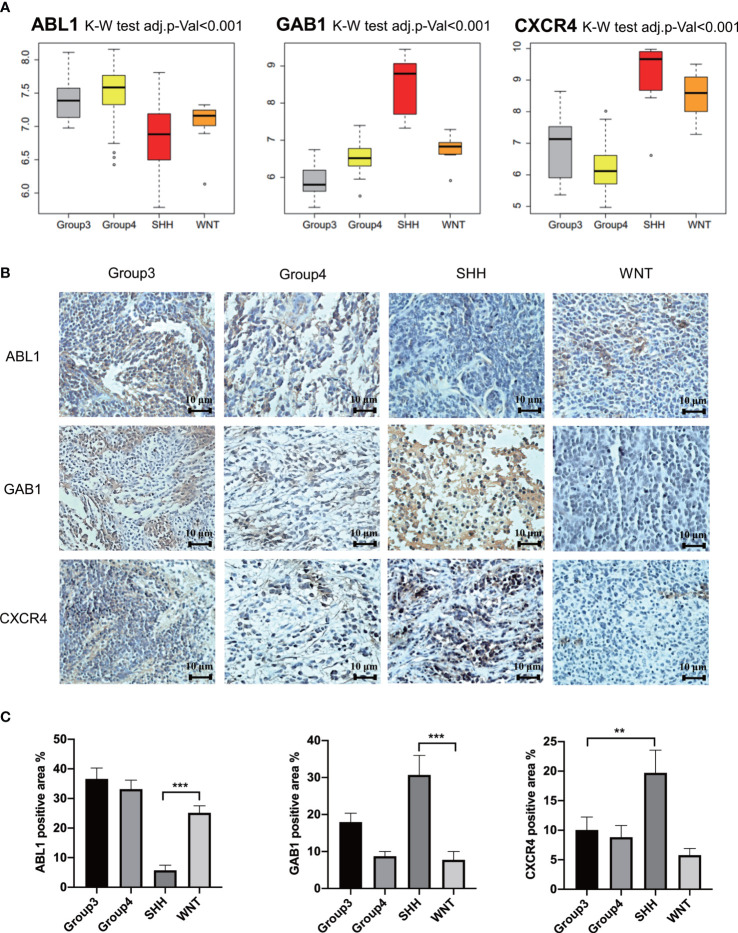
Further verification of hub genes. **(A)** The expression of GAB1, ABL1, and CXCR4 in different subtypes from the independent data set. **(B)** Further verification by immunohistochemistry (n = 5) (** p ≤ 0.01, *** p ≤ 0.001). **(C)** Average positively stained area percentage of ABL1, GAB1, and CXCR4.

GSE37418 was downloaded from GEO databases, which contained 73 MB samples. GSE37418 consisted of 10 samples of SHH subgroup, 8 samples of WNT subgroup, 16 samples of Group 3 subgroup, and 39 samples of Group 4. Comparison and analysis of the expression patterns of subgroup markers were achieved. We found that the expression of GAB1 and CXCR4 was higher in the SHH subtype than those in the three other subtypes, while ABL1 was expressed lower in the SHH subtype and higher in the three other subtypes (**Figure 6A**). Next, the immune hub genes were confirmed by immunohistochemistry (**Figures 6B, C**). The expression of CXCR4 and GAB1 was apparently high in SHH subgroups compared with the other subgroups. As **Figure 6B** illustrates, the expression of ABL1 was lower compared with CXCR4 and GAB1. Results of immunochemistry staining were consistent with the result of our bioinformatic analysis.

### A multifactor regulatory network of medulloblastoma based on immune hub genes

**Figure 7:**
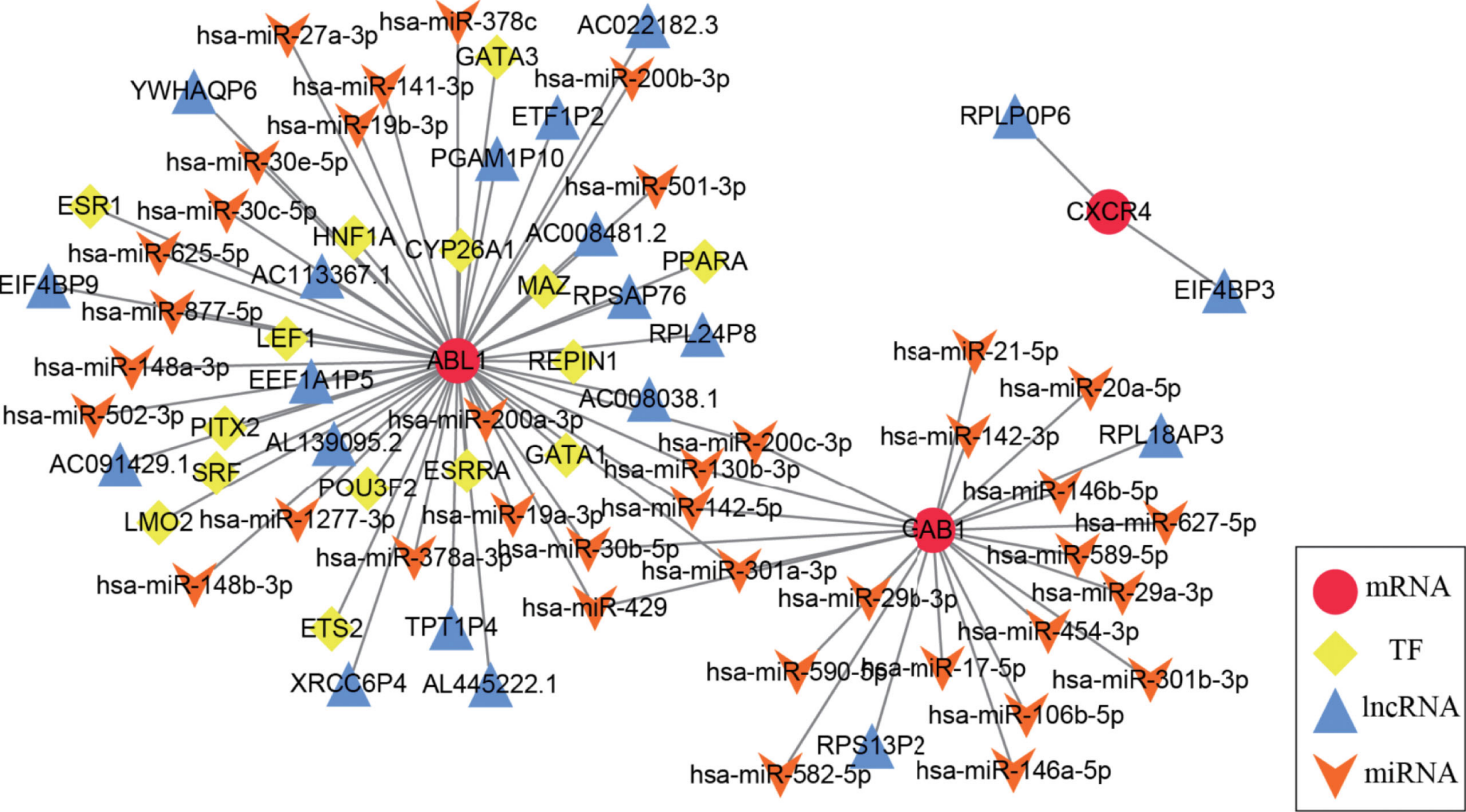
The medulloblastoma multifactor regulatory network is based on immune hub genes. In the multifactor regulatory network of immune hub genes ABL1, GAB1, and CXCR4 in medulloblastoma, each node represents a gene; red circles represent mRNA, yellow diamonds represent TF, blue triangles represent lncRNA, and orange arrows represent miRNA.

A multifactor regulatory network consisting of miRNA, lncRNA, and TF was designed and combined with public databases (such as starBase, Harmonizome) and immune hub genes. Interactions among genes and their following products play a crucial role in many biological processes (25). In the present study, a multifactor network regarding hub genes combined with public databases (such as starBase, and Harmonizome) was designed and constructed, which included miRNA, lncRNA, mRNA, and TF. **Figure 7** shows the multifactor regulatory network of immune hub genes ABL1, GAB1, and CXCR4 in MB. We found that CXCR4 is mainly involved in the regulation of lncRNA. GAB1 is related to the regulation of miRNA. ABL1 is not only involved in the regulation of lncRNA and miRNA but also the regulation of TFs. miRNA, lncRNA, and TF participate in the regulation of gene expression at the transcriptional and posttranscriptional levels and play a crucial role in gene expression. Therefore, they can be used as potential drug targets to indirectly regulate the expression of hub genes by acting on miRNA, lncRNA, or TF.

### The immune hub genes are interrelated with other checkpoint members

**Figure 8:**
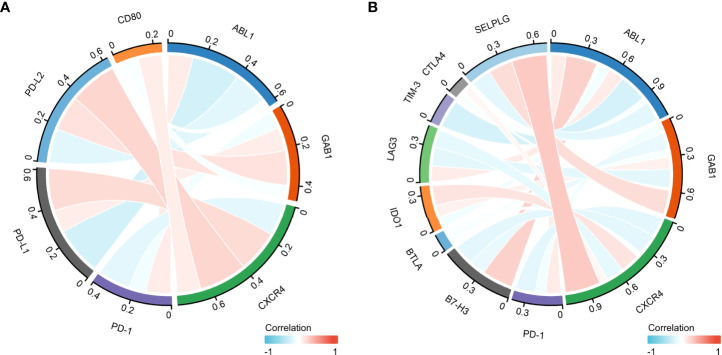
The correlation between the immune hub genes and immune checkpoint genes. **(A)** The correlation between the immune hub genes (ABL1, GAB1, and CXCR4) and the Programmed cell Death 1 (PD-1), Programmed cell Death Ligand 1 (PD-L1) pathway. **(B)** The correlation between the immune hub genes and the other immune checkpoint molecules. Programmed cell Death 1 (PD-1), Programmed cell Death Ligand 1 (PD-L1), Programmed cell Death Ligand 2 (PD-L2), Cluster of Differentiation 80 (CD80), P-selectin glycoprotein ligand-1 (SELPLG), Cytotoxic-T-Lymphocyte-Antigen-4 (CTLA4), T cell immunoglobulin and mucin domain 3(TIM-3), lymphocyte activation gene-3 (LAG3), B- and T-lymphocyte attenuator (BTLA), B7 homolog 3 protein (B7-H3), Indoleamine 2,3-dioxygenase 1 (IDO1), Abelson 1 (ABL1), GRB2-associated binding protein 1 (GAB1), CXC motif chemokine receptor type 4(CXCR4).

Programmed cell death ligand 1 (PD-L1) is one of the ligands of PD-1, and PD-L2 is also engaged in programmed cell death 1 (PD-1) (26). Moreover, CD80, as a costimulatory molecule, can inhibit T-cell activation during the activation stage (27). Thus, we performed Pearson correlation analysis with the expression of the immune hub genes (ABL1, GAB1, and CXCR4), PD-L1, PD-L2, PD-1, and CD80 (**Figure 8A**).

Furthermore, the combination therapy of other oncogenic pathway inhibitors with immune checkpoint blockade therapy has shown clinical benefits (28). Thus, we explored the correlation of other immune checkpoint genes (B7-H3, BTLA, IDO1, LAG3, TIM-3, CTLA4, and SELPLG/PSGL-1) with the immune hub genes (**Figure 8B**).

### The correlation between the immune hub genes and drug sensitivity

**Figure 9:**
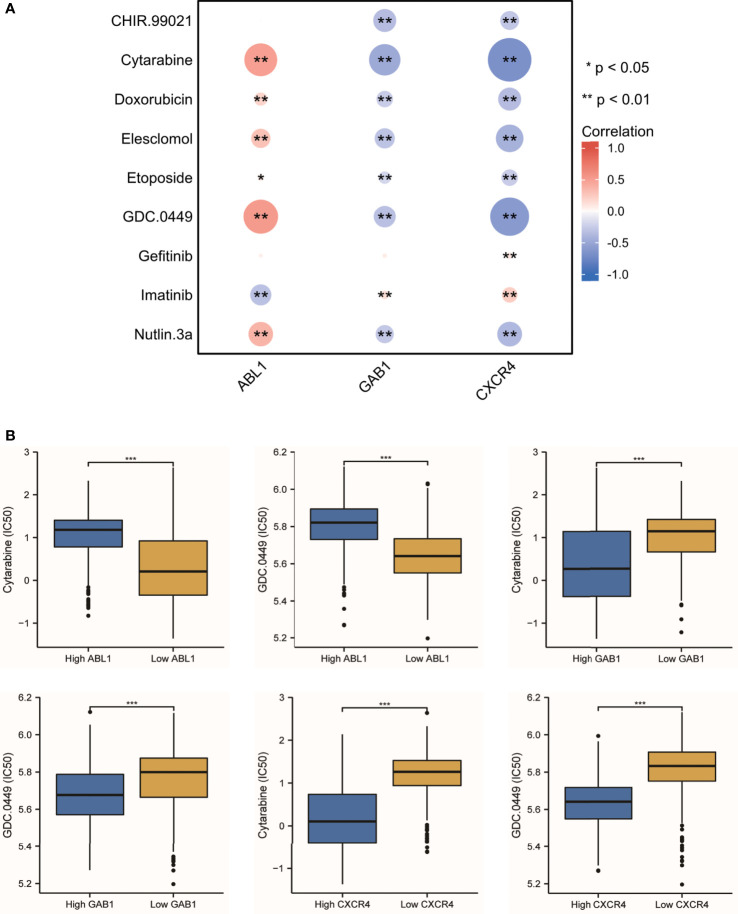
The antitumor drug sensitivity correlated with the immune hub genes. **(A)** Nine antitumor drug sensitivities are correlated with the immune hub genes (* p < 0.05, ** p < 0.001). **(B)** The IC50 of cytarabine and GDC.0449 showed the difference between the high-expression hub gene group and the low-expression hub gene group (*** p ≤ 0.001).

Since the treatment management of MB is stratified and systemic, chemotherapy is often in addition to resection and radiotherapy. The IC50 level of nine antitumor drugs was related to the expression of the immune hub genes (**Figure 9A**). We found that cytarabine and GDC.0449 were closely related to the immune hub genes (**Figure 9B**). Cytarabine was positively correlated with the expression of ABL1 (r = 0.498, p = 3.65E-49), while cytarabine was negatively correlated with the expression of GAB1 (r = -0.476, p = 1.85E-44) and CXCR4 (r = -0.661, p = 5.72E-97). GDC.0449 was positively correlated with the expression of ABL1 (r = 0.524, p = 3.78E-55), while GDC.0449 was negatively correlated with the expression of GAB1 (r = -0.334, p = 2.78E-21) and CXCR4 (r = -0.590, p = 2.78E-21). The IC50s of cytarabine and GDC.0449 were higher in the high ABL1 expression groups than in the low ABL1 expression groups. However, in the high GAB1 and CXCR4 expression groups, the IC50s of cytarabine and GDC.0449 were lower than those in the low GAB1 and CXCR4 expression groups.

## Discussion

MB is the most frequent malignant pediatric brain tumor originating from the posterior fossa, while 30% of cases occur in adults. In the past 20 years, the standard treatment for MB patients is surgery, radiotherapy, and chemotherapy. With the increase in clinical trials of pediatric MB, progression-free survival has gradually improved. However, the treatment for adults and large children with MB needs to be updated urgently (18, 29, 30). The 5-year survival rate was 75%–85%. One of the main complications of surgery is cerebellar reticence. Radiotherapy may cause severe adverse reactions such as cognitive function changes, vascular lesions, and hearing loss. Chemotherapy may have the risk of bone marrow suppression and organ dysfunction. A recent study used SIRT1 and AROS to improve drug resistance in the treatment of neuroblastoma (31). There is an urgent need for more safe and effective adjuvant therapy for MB. Tumor-infiltrating immune cells have been proven to be the criteria for predicting the prognosis of solid tumors and predicting chemotherapy response (32). Thus, the potential of immunotherapy for MB has been discussed for many years (33). However, despite the profound molecular characteristics of MB, the progress of immunotherapy is slow, which may be due to the limited understanding of the tumor microenvironment. Hence, we used the bioinformatic method to quantify eight immune cell populations and two stromal cell populations as well as hub genes in a MB dataset including 763 samples. Our study includes the following aspects: 1) The immune infiltrating cells in each subtype of MB are heterogeneous; 2) Group 3-MBs have a higher presence of immune infiltrative cells (CD8, CD19, and Treg) and endothelial cells compared with those in the other subgroups (**Figure 1B**); 3) Three immune-associated hub genes (ABL1, GAB1, and CXCR4) were identified mostly based on WGCNA algorithm and verified by immunohistochemistry (IHC) technique within patient samples (**Figure 6**); 4) A multifactor regulatory network was built based on the hub genes, miRNAs, lncRNAs, and TFs (**Figure 7**); 5) The immune hub genes are interrelated to immune checkpoint molecules; 6) ABL1 was positively correlated with cytarabine (r = 0.498, p = 3.65E-49) and GDC.0449 (r = 0.524, p = 3.78E-55) sensitivity, while GAB1 (r = -0.476, p = 1.85E-44; r = -0334, p = 2.78E-21, respectively) and CXCR4 (r = -0.661, p = 5.72E-97; r = -0.590, p = 2.78E-21, respectively) were negatively correlated with cytarabine and GDC.0449 sensitivity.

Both transcriptional regulations and posttranscriptional regulations are essential regulatory mechanisms for the cell cycle, including DNA replication, mitosis, and mitotic exit (34). The gene regulatory network (GRN) refers to the network formed by the intercellular gene to gene interaction, and it also refers specifically to the gene-to-gene interaction based on the regulation of gene expression. In addition, more and more research focuses on tumor-infiltrating immune cells and related immunotherapy (33). A previous study showed that a high number of activated cytotoxic T lymphocytes (CTLs) had a worse survival in MB patients (35). Murata et al. (36) suggested that CD8+ tumor-infiltrating lymphocytes (TILs) are protective factors of MB. Using a deconvolution analysis based on methylation expression profiles, we firstly noticed immune infiltrating cells and two stromal cell populations that were statistically different among subgroups. According to the characteristics of the microenvironment, Bockmayr et al. (37) also found significant differences in the aggregation of MB subgroups. Former studies used methods based on expression data such as CIBERSORT, TIMER, and ESTIMATE to approximate the abundance within the immune fraction and scores of enrichments, and they also performed low-resolution deconvolution (38, 39). However, distinguishing cellular subsets and their correlation would be more detailed with more intricate deconvolution using DNA methylation data (23). Thus, MethylCIBERSORT was used first to derive estimates for different immune infiltrating cell populations among subgroups of MB when both DNA methylation and gene expression data can be obtained.

To investigate the immune infiltration of MB, the GO and KEGG enrichment analyses were carried out to explore the biological function of imm-DEGs among subgroups of MB and revealed that the overlapped DEGs were associated with the chemokine signaling pathway. Chemokines and their receptors play an important role in physiological and pathological processes (40). The relationship between chemokines and tumor biology is a subject of great concern in the scientific community. Significant results have been achieved in the treatment of MB by using chemokines and their receptors (41); combined with our analysis, they indicate the important role of chemokines in MB and the possible therapeutic ability. From the perspective of tumor immune infiltration, we have a novel viewpoint in group 3-MBs: group 3-MBs have a grim outcome result from multiple adverse prognostic factors, such as MYC amplification, presence of metastases, and large cell/anaplastic histology (13), while our study indicates the higher presence of endothelial cells and infiltrating immune cells (CD19, CD8, and Treg) in tumor bulk of group 3-MBs compared with the other subgroups. This phenomenon implies that estimating the TILs and endothelial cells can contribute to identifying the progression and prognosis of MB. A previous study showed that the DIME (Differential Methylation Analysis for immune Cell Estimation)-TIL score was a negative prognostic factor in MB (42), which laterally supported our hypothesis. Furthermore, tumor endothelial cells are required for tumor angiogenesis, which is crucial for tumor progression and metastasis (43). Thus, our studies reveal that the tumor immune and tumor endothelial cell infiltration may play a role in pathogenesis underlying the group 3-MBs, which is important to control this fatal subtype of MB and provide a basis for immunotherapy.

The module identified by WGCNA is highly related to a certain personality, and the genes that make up this module are likely to participate in a certain pathway or biological process; through the construction of the PPI network and the identification of the hub gene, we can further screen out some genes that may play a key role in this process to provide more accurate identification of subtype markers (22); therefore, WGCNA was used to identify the gene modules that are specifically associated with similar expression patterns in MBs. A total of 294 imm-DEGs were investigated and categorized into five modules. The blue module was chosen as the key module and included in the construction of the PPI network based on its significant correlation with the trait of immune infiltration. According to the results of the blue module and the PPI network, we have identified three hub genes, CXCR4, GAB1, and ABL1, as immune infiltration markers among subgroups of MB; a multifactor regulatory network was also set up based on the three hub genes.

Sengupta et al. (44) found that CXCR4 only exhibited overexpression in the SHH-MBs when compared with normally high levels found in the fetal cerebellum, and it was essential for SHH pathway activation in MB. It has been demonstrated by Yang et al. (45) that the inhibition of CXCR4 blocked the intracranial growth of MB cell lines. Similarly, Ward et al. (46) combined CXCR4 and SHH pathway antagonism, which produced an enhanced therapeutic effect. In the present study, CXCR4 was significantly highly expressed in SHH-MBs compared with the three other subgroups, which is encouraging, as this confirmed the close relationship between CXCR4 and SHH at the transcriptional level. Also, CXCR4 was associated with the immune response of other tumors, and anti-CXCR4 union and anti-PD-1 immunotherapies are used in treating other CNS tumors, which further confirmed that CXCR4 is related to immune infiltration (47). GAB1, a member of the GAB/DOS family of adapter proteins, is tyrosine-phosphorylated when stimulated by various growth factors and cytokines (48). Immunoreactivity for GAB1 was found specific in SHH-MBs in a cohort (n = 235) of MB patients aged between 0.4 and 52 years and related to a worse survival outcome (49). Although the role of GAB1 in the SHH pathway in MBs and other central system tumors is still not elucidated, the current study discovered a higher expression of GAB1 in SHH-MB compared with the other subgroups, which is consistent with the above literature. ABL1, a non-receptor tyrosine kinase, is involved in various biological functions. Zhu et al. (50) identified that ABL1 with 11 other genes can construct a prognostic nomogram predicting the overall survival for MB patients. In our study, ABL1 was initially thought to be expressed lowly in SHH-MB, while high in the three other subgroups.

Unfortunately, the present study has not explored the multifactor regulatory network constructed by the hub gene in-depth. This is due to the lack of clarification on the mechanism of the three hub genes, leaving us room for further investigation.

In conclusion, the present study has proposed bioinformatic results of the imm-DEGs, three immune hub genes (CXCR4, GAB1, ABL1), and the multifactor regulatory network from datasets of GEO and immune gene profiles by algorithms (melthyCIBERSORT, WGCNA), PPI network, and public databases (starBase, Harmonizome). The three immune hub genes with differential expressions among subgroups of MB emphasize the heterogenicity of the tumor immune infiltration microenvironment in MB subgroups. With the rapid development of high-throughput analysis, molecular stratifications are gradually becoming the main trend in molecular targeted therapy and prognostic risk assessment model. Our research is just a step toward exploring tumor immune infiltration and provides the framework for the identification of immune infiltration markers and multifactor regulatory networks in MBs. The analysis based on immune differences has important clinical significance for molecular typing and immunotherapy. However, there are still some limitations in this study. Transcriptome analysis only reflects some aspects of the immune state rather than the overall changes. The number of samples for verification *via* immunohistochemistry is limited, since the restrictions on obtaining patient samples. We will further explore this in the follow-up study.

## Data availability statement

The original contributions presented in the study are included in the article/**Supplementary material**. Further inquiries can be directed to the corresponding author.

## Ethics statement

This study was approved by the Institutional Review Boards of Beijing Tiantan Hospital (Beijing, China). The procedures of the human tissues collection were reviewed and approved by the Ethics Committee of Beijing Tiantan Hospital, Capital Medical University [approval no. 2021-020-01]. The patients/participants provided their written informed consent to participate in this study.

## Author contributions

WL and FC were involved in conceptualization. SL designed the research and guided the experiments. CW, LP, XZ, and ZC were involved in analyzing and interpreting the data. SL and JC were involved in the experimental validation. JC was involved in formal writing. SL and FC were involved in review and editing. FC was involved in supervision. WL was involved in funding acquisition. All authors contributed to the article and approved the submitted version.

## Funding

This research was funded by The National Natural Science Foundation of China, grant number 81972338. This study was also funded by Advanced Research and Training Program of Beijing Double Leading Scholars from China academy of Chinese Medical Science. This research was also supported by Clinical Major Specialty Projects of Beijing and Beijing Advanced Innovation Center for Big Data-based Precision Medicine, Capital Medical University, Beijing, 100069, China.

## Acknowledgments

We would like to thank China National Clinical Research Center for Neurological Diseases and Chinese Medical Science for administrative support.

## Conflict of interest

The authors declare that the research was conducted in the absence of any commercial or financial relationships that could be construed as a potential conflict of interest.

## Publisher’s note

All claims expressed in this article are solely those of the authors and do not necessarily represent those of their affiliated organizations, or those of the publisher, the editors and the reviewers. Any product that may be evaluated in this article, or claim that may be made by its manufacturer, is not guaranteed or endorsed by the publisher.

## Abbreviations

MB, medulloblastoma; WNT, wingless; SHH, sonic hedgehog; TIME, tumor immune microenvironment; CAR-T, chimeric antigen receptor T cell; GEO, Gene Expression Omnibus; GO, Gene Ontology; KEGG, Kyoto Encyclopedia of Genes and Genomes; WGCNA, weighted correlation network analysis; MM, module membership; PPI, protein–protein interaction; STRING, Search Tool for the Retrieval of Interacting Genes; TF, transcriptional factor; imm-DEGs, differentially expressed immune genes; GRN, gene regulatory network; CTLs, cytotoxic T lymphocytes; GAB1, GRB2-associated-binding protein 1; CXCR4, CXC motif chemokine receptor type 4; ABL1, Abelson 1.
