# Supplementary material for: Molecular profile reveals immune-associated markers of medulloblastoma for different subtypes
Source: Front Immunol. 2022 Jul 28;13:911260. doi: 10.3389/fimmu.2022.911260 (PMC9367478; doi:10.3389/fimmu.2022.911260)
Supplement: Supplementary file 1 [file DataSheet_1.docx]

**Molecular profile reveals immune-associated markers of medulloblastoma for different subtypes**

Jinyi Chen^1#^, Zhuang Kang^1#^, Shenglan Li^1^, Can Wang^1^, Xiaohong Zheng, Zehao Cai^1^, Leixin Pan^2^, Feng Chen^1*^and Wenbin Li ^1*^

^1^Department of Neuro-oncology, Cancer center, Beijing Tiantan Hospital, Capital Medical University, Beijing, 100070, China.

^2^School of Mechatronical Engineering, Beijing Institute of Technology, Beijing, 100081, P.R. China.

***Corresponding Author:** Wenbin Li, MD

Department of Neuro-oncology, Cancer Center, Beijing Tiantan Hospital, Capital Medical University, Beijing, China.

**Address:** NO.119, Nansihuan West Road, Fengtai District, Beijing, China.

**Postal Code:** 100070. **Email:** [liwenbin@ccmu.edu.cn](mailto:liwenbin@ccmu.edu.cn). **Phone:** 86015301377998

***Corresponding Author:** Feng Chen, MD

Department of Neuro-oncology, Cancer Center, Beijing Tiantan Hospital, Capital Medical University, Beijing, China.

**Address:** NO.119, Nansihuan West Road, Fengtai District, Beijing, China.

**Postal Code:** 100070. Email: [chenfeng@bjtth.org](mailto:chenfeng@bjtth.org). Phone: 86018500097676

Keywords: MethylCIBERSORT, immune infiltration, WGCNA, PPI, Medulloblastoma.

Supplementary Table I. Detailed information of tissue microarrays

| No. | Age(years) | Sex | Pathology diagnosis | Subtypes |
| --- | --- | --- | --- | --- |
| 1 | 32 | Female | Medulloblastoma (cerebellum) | SHH |
| 2 | 53 | Male | Medulloblastoma (cerebellum) | SHH |
| 3 | 41 | Male | Medulloblastoma (cerebellum) | SHH |
| 4 | 49 | Female | Medulloblastoma (cerebellum) | SHH |
| 5 | 34 | Female | Medulloblastoma (cerebellum) | SHH |
| 6 | 8 | Female | Medulloblastoma (ventricle) | WNT |
| 7 | 9 | Male | Medulloblastoma (cerebellum) | WNT |
| 8 | 12 | Female | Medulloblastoma (cerebellum) | WNT |
| 9 | 4 | Female | Medulloblastoma (cerebellum) | WNT |
| 10 | 6 | Male | Medulloblastoma (ventricle) | WNT |
| 11 | 25 | Male | Medulloblastoma (fourth ventricle) | Group3 |
| 12 | 11 | Male | Medulloblastoma (fourth ventricle) | Group3 |
| 13 | 17 | Male | Medulloblastoma (cerebellum) | Group3 |
| 14 | 5 | Female | Medulloblastoma (cerebellum) | Group3 |
| 15 | 4 | Male | Medulloblastoma (cerebellum) | Group3 |
| 16 | 17 | Male | Medulloblastoma (cerebellum) | Group4 |
| 17 | 5 | Female | Medulloblastoma (fourth ventricle) | Group4 |
| 18 | 3 | Male | Medulloblastoma (cerebellum) | Group4 |
| 19 | 7 | Female | Medulloblastoma (cerebellum) | Group4 |
| 20 | 16 | Male | Medulloblastoma (vermis) | Group4 |


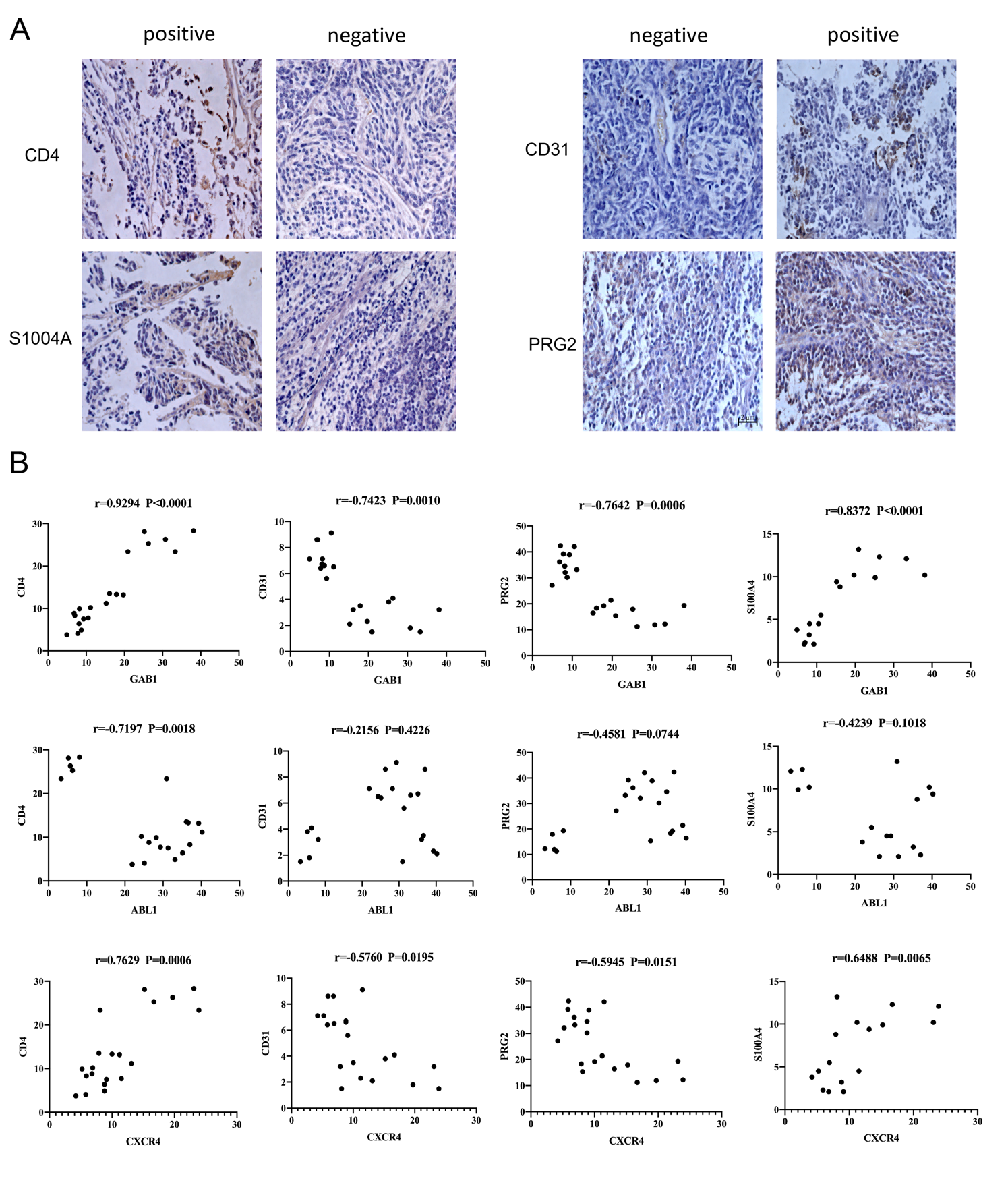


Supplementary Figure 1 (A) Immunohistochemistry of CD31, CD4, PRG2 and S100A4. (B) The correlation between hub genes (GAB1, ABL1, CXCR4) and immune infiltrating cells (CD4_Eff, Fibroblast cells, Endothelial cells and the Eos cells) by immunohistochemistry. There are 5 samples in each group (SHH, WNT, Group 3, Group 4).


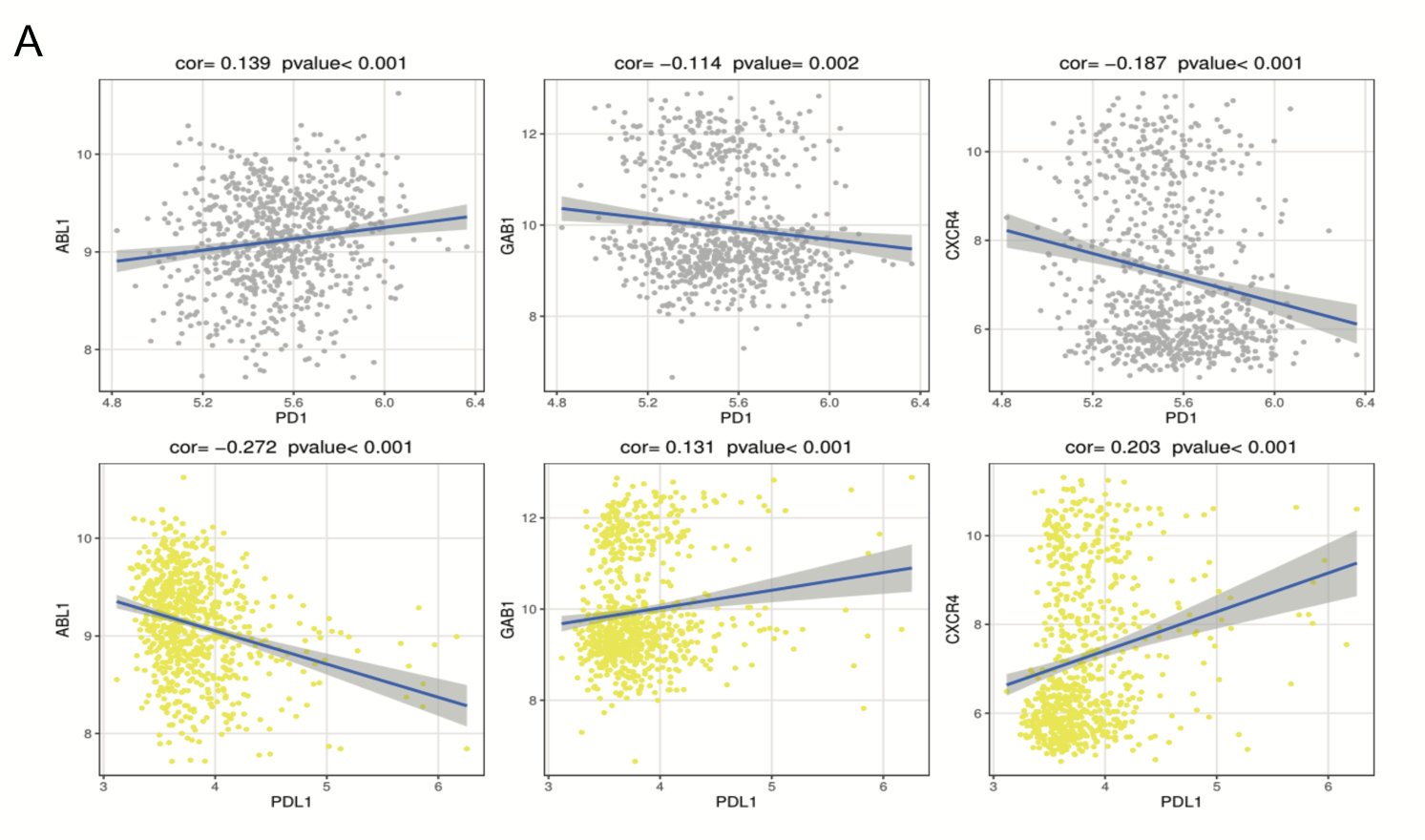


Supplementary Figure 2. Correlation between hub gene and immune checkpoint such as PD1 and PD-L1.
