# Supplementary material for: Molecular profile reveals immune-associated markers of medulloblastoma for different subtypes
Source: Front Immunol. 2022 Jul 28;13:911260. doi: 10.3389/fimmu.2022.911260 (PMC9367478; doi:10.3389/fimmu.2022.911260)
Supplement: Supplementary file 2 [file DataSheet_2.docx]

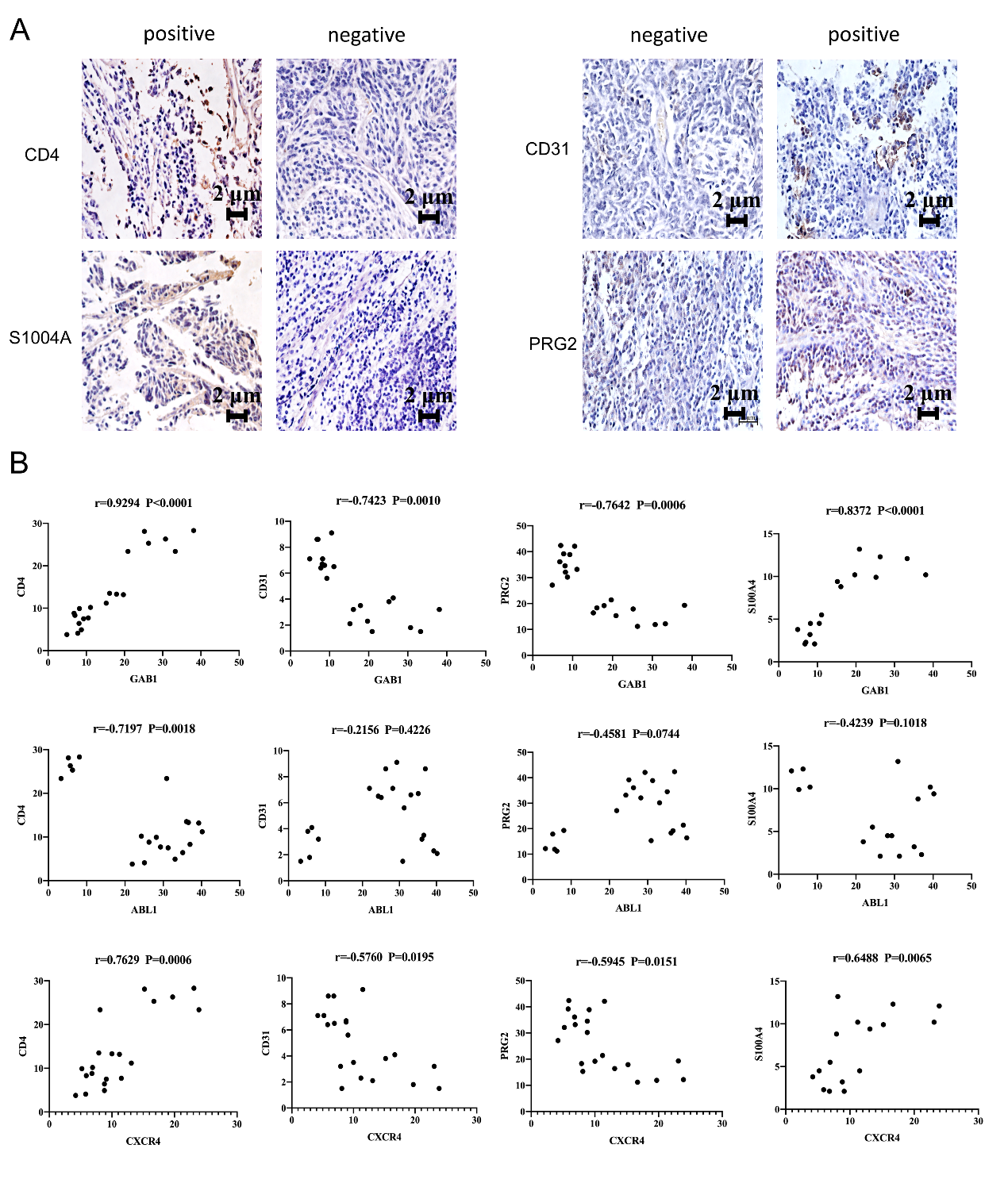


Supplementary Figure S1. The correlation between the three immune hub genes and the marker of immune cells. (A). The validation of the marker of the immune cell by IHC in 20 patients’ paraffin samples. (B) The correlation between the three immune hub genes and the marker of immune cells. (Pearson correlated analysis.)


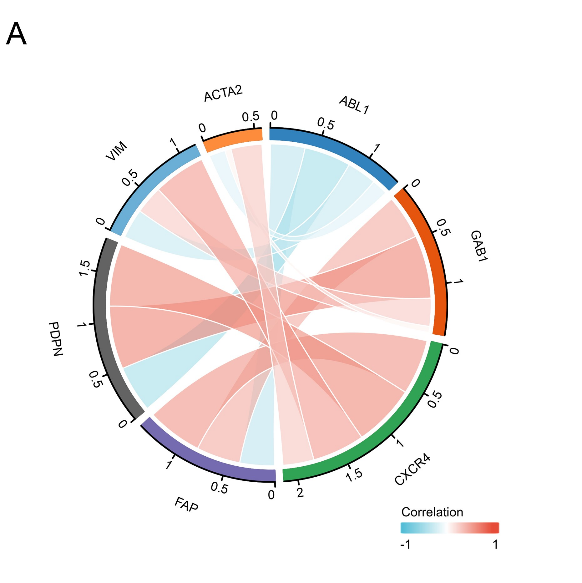


Supplementary Figure S2. The correlation between the three immune hub genes and the markers of cancer-associated fibroblasts. The expression of ABL1 is negatively correlated to the expression of the marker genes (ACTA2, r=-0.172, p= 1.86E-06; VIM, r=-0.314, p=6.80E-19, PDPN, r=-0.495, p=2.06E-48; FAP, r=-0.367, p=8.35E-26) of CAFs. The expression of GAB1 is positively correlated to the expression of the marker genes (ACTA2, r=0.064, p=0.046; VIM, r=0.298, p=4.00E-17, PDPN, r=0.670, p=1.39E-100; FAP, r=0.467, p=1.44E-42) of CAFs. The expression of CXCR4 is positively correlated to the expression of the marker genes (ACTA2, r=0.329, p=7.88E-21; VIM, r=0.555, p=5.77E-63, PDPN, r=0.659, p=1.50E-96; FAP, r=0.588, p=4.06E-72) of CAFs.
